# Dental Wear: Attrition, Erosion, and Abrasion—A Palaeo-Odontological Approach

**DOI:** 10.3390/dj5020019

**Published:** 2017-06-17

**Authors:** Geoffrey H. Sperber

**Affiliations:** Faculty of Medicine & Dentistry, University of Alberta, Edmonton, AB T6G 1C9, Canada; gsperber@ualberta.ca

**Keywords:** tooth wear, attrition, erosion, abrasion, palaeo-odontology

## Abstract

This paper reviews the surface ablation of early hominin teeth by attrition, abrasion, and erosive dental wear. The occurrence of these lesions is explored in a sample of South African fossil australopithecine dentitions revealing excessive wear. Interpretation of the nature of the dietary components causing such wear in the absence of carious erosion provides insight into the ecology of the Plio-pleistocene epoch (1–2 million years ago). Fossil teeth inform much of the living past by their retained evidence after death. Tooth wear is the ultimate forensic dental evidence of lives lived.

## 1. Introduction

The wear of teeth is an inevitable consequence of their evolutionary designated purpose of food acquisition by predation, apprehension, grasping, trituration, mastication, and ingestion. The enormous variety of shapes, sizes, and locations of teeth encountered in comparative odontology attests to the innovative evolutionary outcomes of obtaining sustenance for survival. The mechanism of odontogenesis, whereby enamel amelogenesis is completed by the demise of ameloblasts before dental eruption, ensures that no repair mechanism exists for the post-eruption repair of worn or damaged enamel. Accordingly, any occurrence of damage of whatever source is permanently imprinted upon the hardest and most prolonged remains of creatures possessing teeth. The study of the impingement upon teeth by different elements constitutes the purpose of this investigation.

Dental attrition is caused by tooth to tooth contact forming acquired wear facets upon pristine enamel, whereas abrasion is caused by food and foreign body contact (e.g., tooth brushing) that may obliterate attrition wear patterns. Finally, erosion by acid-based leaching and dissolution of the hydroxyapatite crystals of enamel may override previous lesions of enamel. Accordingly, critical analysis of the causes and history of damaged enamel needs to be undertaken in identifying its initial origin. Tooth wear is thus multifactorial in origin, based upon diet and eating habits, oral hygiene, bruxism, brushing habits, xerostomia, anorexia, gastro-esophageal reflex disease (GERD), vomiting, bulimia as well as medications and dietary supplements. A need for standardization and classification of these multiple determinants of dental deterioration has been proposed [1]. Dental surface texture analysis provides evidence of the etiological factors implicated in attrition and erosion [2].

## 2. Etiology of Dental Wear

The phenomenon of dental wear is specifically allied to both clinical dentistry and to comparative odontology and palaeo-anthropology. The patterns of wear upon teeth become at once both a clinical concern in current dental practice, of interest in forensic odontology, and the subject of exploration in palaeo-odontology. The occlusal wear pattern created in ideal contemporary dentitions reveals an antero-posterior curvature of the mandibular teeth first described by Ferdinand Graf von Spee [3,4]. By contrast, the curve of Wilson observed in the coronal plane is an upward U-shaped curvature of the maxillomandibular occlusal plane. These attritional wear planes are dependent upon the main masticatory forces exerted by the masseter and temporalis muscles that provide the most favorable loading forces upon the teeth. Thus, attritional tooth wear must be considered a physiological phenomenon, creating the Monson curve of occlusion. This normal wear pattern must be contrasted with pathological abrasive wear caused by bruxism, jaw clenching, pipe-clenching, oral musical instrument usage, bottle cap opening and the use of teeth for gripping objects. Dental erosion is an expression of carious pathological lesions or acidic agents leaching dental tissues.

Different categories of teeth wear at different rates. Thus, incisors suffer the greatest wear (97%), followed by the molars (85%), then the canines (74%), with the premolars the least worn (60–68%) [5]. 

## 3. Enamel Thickness

As enamel is the tissue most subject to wear, the hardness and thickness of enamel become evolutionary responses to ecological and dietary variations. Thus, the thickened enamel of Australopithecine dentitions contrasts sharply with the thin enamel of orangutans (*Pongo pygmaeus*), chimpanzees (*Pan troglodytes*) and gorillas, reflecting their dietary habits. Much can be inferred from varying enamel thicknesses [6,7].

Dental microwear patterns reveal both occlusal dynamics and the nature of the diets and the properties of the procured and ingested food. Scanning electron microscopy and stable isotopes can identify specific patterns of enamel wear related to particular diets or tooth use behavior [8]. The characteristics of microwear provide implications for hominin diets [9]. 

**Figure 1:**
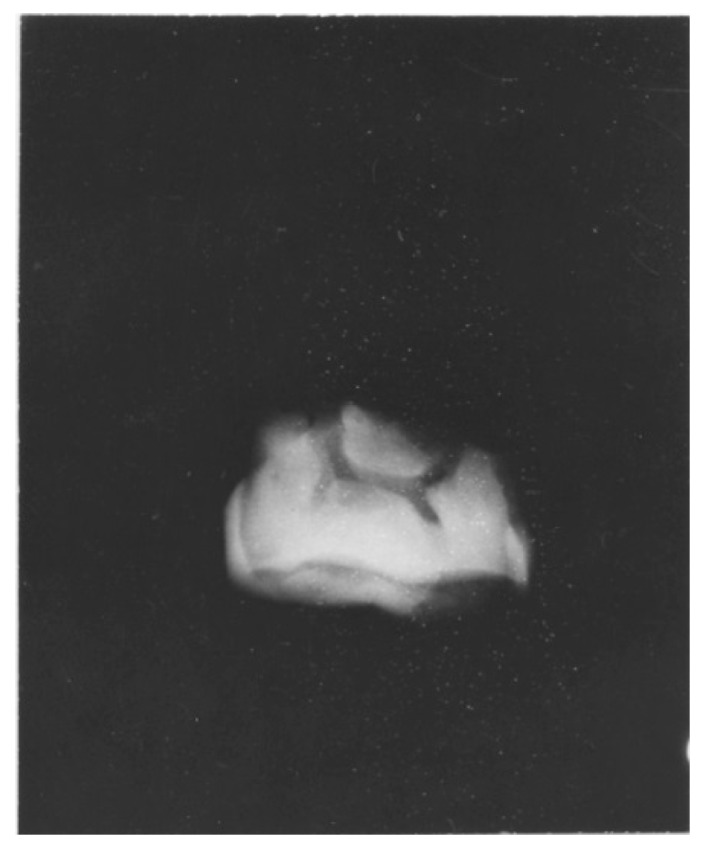
Australopithecus africanus. StW Hom 1/68. Radiograph of isolated maxillary crown. The buccal and lingual edges of the worn occlusal surface are seen as wavy lines reflecting the scalloped occlusal table. The severe wear impacted the underlying dentine, resulting in a secondary dentine deposition in the pulp chamber. The roots of this specimen were broken off.

Enamel structure by prismatic weaving, limiting stress fracturing, causes differential resistance to wear, effectively sculpting occlusal surfaces by variable abrasive patterns. Prism orientation in enamel can direct abrasion and attrition to form ridges or sharp edges for trituration efficiency (Figure 1).

The pattern of attritional wear plays a significant role in interpreting the nature of dietary materials. The palaeo-eocology of extinct hominins can be elicited from dental microwear and isotopic analysis of enamel. In this respect, the degree and wear patterns of fossil hominid dentitions are revealing of past ecological habitats. The folivorous diet of the great apes, relatively clean of abrasive grit, is not as wearing on dental enamel as the presumed gritty omnivorous diet (roots, sedges, meat and bones) of australopithecines and modern man. The selective dietary pressures for thicker enamel are evident in the greater longevity of hominids, including man, compared to the briefer lives of thin enameled pongids.

## 4. Australopithecine and Paranthropus Dentitions

The australopithecines existed between three and two million years ago in various African fossil sites. The distinctions drawn between “gracile” (*Australopithecus africanus*) and “robust” australopithecines (*Paranthropus robustus*) are based not only on differences in gross morphology, but also on their dentitions [10,11,12]. The variation in dental microwear exhibited between these species leads to inconclusive determination of the dietary causative factors of their tooth wear patterns. Differential isotopic evidence of dental components further compounds the variability of dietary intake [13].

**Figure 2:**
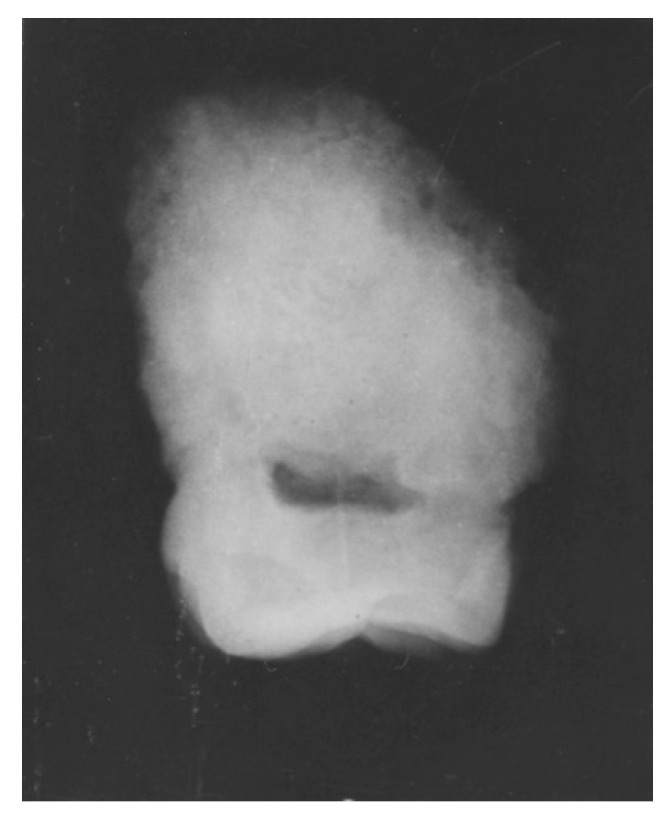
Australopithecus africanus StW Hom 2/69. Radiograph of isolated maxillary M3 crown.

**Figure 3:**
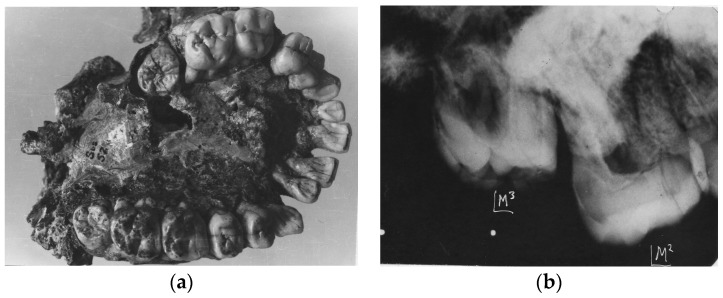
(**a**) Australopoithecus africanus Sts 52(a). Occlusal view of the complete maxillary dentition. Warping of the maxilla has distorted the dental arcade. Moderate occlusal attrition of the M1 molars contrasts with the slight wear on the later erupted M2s and the pristine unerupted M3s; (**b**) Australopithecus africanus Sts 52(a). X-ray of left maxillary M2 and unerupted M3. The unworn M3 contrasts with the slight distal wear on M2.

**Figure 4:**
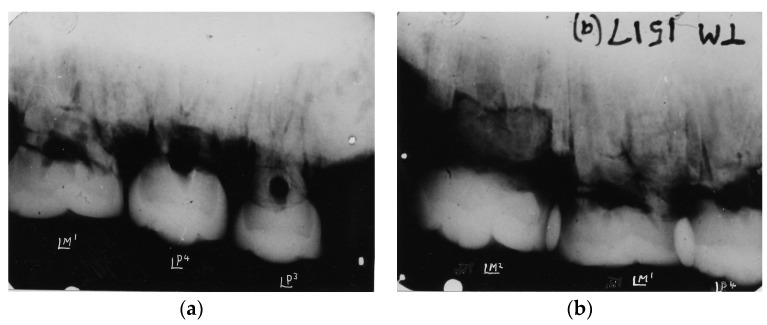
(**a**) Kromdraai Paranthropus TM 1517(a). Radiograph of left premolar and molar maxillary teeth reveal the graded wear of the premolars and M1; (**b**) Kromdraai Paranthropus TM 1517(a). Radiograph of left maxillary second premolar and maxillary M1 and M2 revealing heavy wear distally on the premolar and occlusal surface of M1 and slight wear mesially on M2.

**Figure 5:**
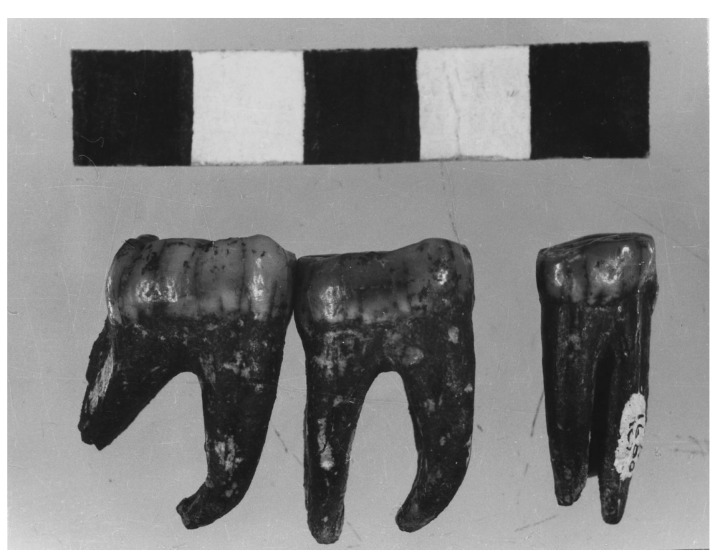
Kromdraai Paranthropus TM 1600. Isolated mandibular premolar, 1st and 2nd mandibular molars exhibiting severe abrasive wear, attesting to a rough diet.

**Figure 6:**
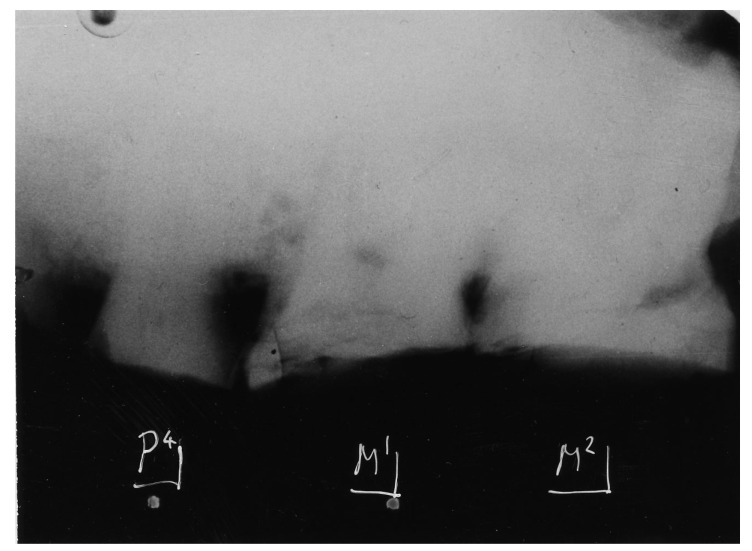
Australopithecus africanus Makapansgat MLD 9. Radiograph of maxillary teeth in situ exhibiting extreme abrasive wear of P4, M1 and M2, attesting to a tough abrasive diet early in life.

**Figure 7:**
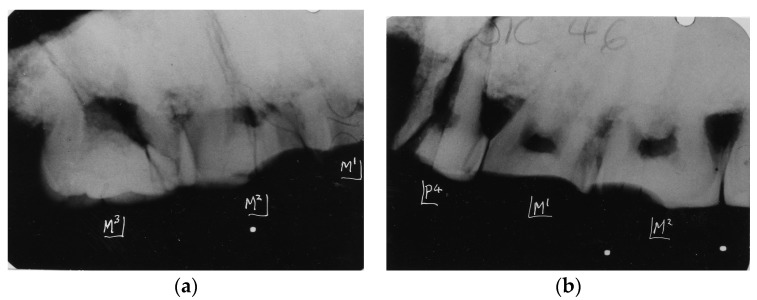
(**a**) Paranthropus robustus. Kromdraai. SK 46. Radiograph of right maxillary teeth revealing graded wear from M1 (severe) to minor wear on M3; (**b**) Paranthropus robustus. Kromdraai. SK 46. Radiograph of left maxillary teeth. An unusual pattern of severe wear confined to the mesial half of M2 is seen that requires some explanation. The less worn distal half of this tooth and M3 suggests absence of the corresponding occluding mandibular M2 and M3. Thereby, unusual wear patterns can reveal peculiar phenomena of abrasive wear.

The following Australopithecine dentitions exhibit varying degrees of attritional and abrasion wear (Figure 1, Figure 2, Figure 3, Figure 4, Figure 5, Figure 6 and Figure 7). The images portrayed are from original photographs and X-rays taken personally in 1970–1972 at the University of the Witwatersrand, Johannesburg, South Africa as part of a PhD thesis [14].

## 5. Conclusions

Distinguishing between abrasion and attrition in early hominin dentitions might provide evidence of the nature of dietary components. However, the examples provided here of extreme wear in the comparatively short lifespans of the australopithecines indicate very abrasive diets. Significantly, no evidence of erosive impact characteristic of dental caries was found in any of the specimens examined.

The information that can be gleaned from tooth wear is subject to many interpretations of their etiology by different agents over a lifetime of dental usage. Teeth tell tales of diets and lives lived long after death prevails, revealing much but not all of the history of the food ingestion experienced by the creatures studied.
